# Anatomically Discrete Sex Differences in Neuroplasticity in Zebra Finches as Reflected by Perineuronal Nets

**DOI:** 10.1371/journal.pone.0123199

**Published:** 2015-04-07

**Authors:** Gilles Cornez, Sita M. ter Haar, Charlotte A. Cornil, Jacques Balthazart

**Affiliations:** GIGA Neurosciences, Research group in Behavioral Neuroendocrinology, University of Liège, Liège, Belgium; Utrecht University, NETHERLANDS

## Abstract

Large morphological sex differences in the vertebrate brain were initially identified in song control nuclei of oscines. Besides gross differences between volumes of nuclei in males and females, sex differences also concern the size and dendritic arborization of neurons and various neurochemical markers, such as the calcium-binding protein parvalbumin (PV). Perineuronal nets (PNN) of the extracellular matrix are aggregates of different compounds, mainly chondroitin sulfate proteoglycans, that surround subsets of neurons, often expressing PV. PNN develop in zebra finches song control nuclei around the end of the sensitive period for song learning and tutor deprivation, known to delay the end of the song learning sensitive period, decreases the numbers of PNN in HVC. We demonstrate here the existence in zebra finches of a major sex difference (males > females) affecting the number of PNN (especially those surrounding PV-positive cells) in HVC and to a smaller extent the robust nucleus of the arcopallium, RA, the two main nuclei controlling song production. These differences were not present in Area X and LMAN, the lateral magnocellular nucleus of the anterior nidopallium. A dense expression of material immunoreactive for chondroitin sulfate was also detected in several nuclei of the auditory and visual pathways. This material was often organized in perineuronal rings but quantification of these PNN did not reveal any sex difference with the exception that the percentage of PNN surrounding PV-ir cells in the dorsal lateral mesencephalic nucleus, MLd, was larger in females than in males, a sex difference in the opposite direction compared to what is seen in HVC and RA. These data confirm and extend previous studies demonstrating the sex difference affecting PNN in HVC-RA by showing that this sex difference is anatomically specific and does not concern visual or auditory pathways.

## Introduction

Brain plasticity was initially considered only at the functional level and covered phenomena such as long-term potentiation or depression (LTP/LTD)[1]. It was however realized that there is also a morphological plasticity in the brain including eventually addition of new neurons in specific structures throughout the entire life [2]. Understanding this plasticity in aging, during neurodegenerative diseases or after injury is of paramount biomedical and societal interest and the analysis of model systems where such a plasticity is spontaneously displayed seasonally or as a function of experience might provide important insights into underlying mechanisms. Changes in neuronal connectivity represent an important part of this morphological plasticity. They are controlled by a variety of mechanisms including changes in the extension of glial cells, secretion of extracellular matrix proteins and activity of a variety of enzymes.

Songbirds such as zebra finches (*Taeniopygia guttata*) learn their song by processes that present significant analogies with the acquisition of human speech; they are thus useful models to study experience-dependent plasticity. Both songbirds and humans need a tutor to learn their species-specific vocalizations and go through similar developmental phases [3–5]. Song learning in male zebra finches takes place during a sensitive period that ends at about 90 days post-hatch. During this period, juveniles need to hear male adult song as a model. This song will be memorized during the sensory phase and this memorized template will be compared to first imperfect songs produced during the sensori-motor phase (subsong, equivalent to babbling in children) before the fully stable crystallized song develops [2,3,5,6]. Tutor deprivation or deafening during ontogeny leads to less structured and unstable song in adulthood [3,7,8].

In zebra finches only males sing whereas females never do so even if treated with exogenous testosterone in adulthood [9,10]. This major qualitative behavioral difference correlates with extensive neuroanatomical differences affecting a set of discrete brain nuclei that control the learning and production of song, the so-called song system [11]. Two main pathways have been identified in the song system. The posterior motor pathway connects HVC (used as a proper name; see [12]) to the robust nucleus of the arcopallium (RA) that ultimately projects to the motoneurons innervating the syrinx. This pathway clearly controls song production and lesions of HVC or RA block song production [13–15]. The anterior forebrain pathway also links HVC to RA but indirectly via Area X of the basal ganglia, the medial part of the dorsolateral thalamic nucleus (DLM) and the lateral magnocellular nucleus of the anterior nidopallium (LMAN) (see [16] for a review). Lesions of this pathway in adulthood do not disrupt singing in the short term but lesions during ontogeny markedly inhibit song learning. This pathway also seems to be implicated in the control of song stability [5,17–19].

Multiple sex differences in the song control system have been described in association with the absence of singing behavior in female zebra finches. They concern the volume of most nuclei that are larger in males than in females, with Area X being completely absent in females, the numbers, size and dendritic arbor of neurons in these nuclei and the connections between them [11,20,21]. Connections between neurons of the song control system are largely established during ontogeny (see [22] for a detailed analysis of the growth of the HVC to RA projection) but they remain plastic to some extent in adulthood at least in canaries and starlings, two open-ended learner species [23–25]; to our knowledge, this type of plasticity has not been reported in zebra finches. This plasticity involves retraction and regrowth of axonal/dendritic processes associated with an important reorganization of the neuronal extracellular matrix that limits intercellular connections. Such a reorganization of the extracellular matrix might also occur in a sexually differentiated manner during zebra finch ontogeny when neural connections are formed and stabilized.

Perineuronal nets (PNN) of the extracellular matrix are aggregates of different compounds, mainly chondroitin sulfate proteoglycans that surround subsets of neurons, mostly GABAergic inhibitory interneurons [26–28]. These neurons often express parvalbumin (PV), a calcium binding protein, and PV-positive cells are thus frequently surrounded by PNN.

The specific role of PNN remains somewhat unclear but they seem to prevent plasticity through stabilization of the extracellular milieu and synaptic stabilization. Their implication in experience-dependent neural plasticity has been extensively studied in the mammalian visual cortex where it was demonstrated that degradation of PNN with chondroitinase ABC restores visual plasticity [27–29]. Similarly enzymatic degradation of chondroitin sulfate with chondroitinase ABC promotes axonal growth and functional recovery after spinal cord injury [30]. In mouse cell cultures, chondroitin sulfate degradation leads to higher GABAergic excitability [31]. In general, the development of PNN appears to be associated with a decrease in synaptic plasticity and the end of sensitive periods in multiple models of neural plasticity.

In zebra finches specifically, PV neurons have a high electrophysiological activity during early phases of development associated with song learning. Their electrophysiological activity diminishes at later stages when they have been shown to be surrounded by PNN [32]. PV neurons are more numerous in vocal learner than in vocal non-learner species in both mammals and birds. It has thus been suggested that they could be implicated in song and vocal behavior development [33], a notion consistent with the observation that these neurons appear early during the sensitive period for song learning in zebra finches. In Area X specifically, a brain region relevant for song acquisition, the number of PV neurons is higher in 33 day-old than in adult males [8].

In contrast, the end of the sensitive period for song learning seems to be temporally associated with the development of PNN in the song control nuclei HVC and RA so that PNN are more numerous in adult than in 33-day-old males and they are more frequently associated with PV-positive neurons [8]. Most interestingly tutor deprivation (social isolation), which is known to delay the closure of the song learning sensitive period, decreases the percentage of PNN surrounding PV-positive cells and the percentage of PV-positive neurons in HVC [8], clearly suggesting that these structures play a role in the sensitive phase of song learning and stabilization of the related neural structures as shown in other functional systems [28,34]. In one recent set of experiments, a chondroitinase ABC treatment alone failed to restore song plasticity in adult zebra finches but did so when associated with social interactions [35].

Although female zebra finches do not sing they must learn to recognize male song. The song control system, in particular HVC, seems to be implicated in this process in songbirds and it has been shown that lesions of HVC or LMAN in female songbirds modified their ability to discriminate male songs [36–38] (but see [39] suggesting a lack of effect of HVC lesions in zebra finches). Other studies have however suggested that auditory brain areas, especially the caudal medial nidopallium (NCM) in males and the caudomedial mesopallium (CMM) in females, are critical for the storage of the learned information related to the species-specific male song [40–42]. One can thus hypothesize that the neural pathways mediating these processes develop differentially in male and female zebra finches and must be sexually differentiated in adult birds. Indeed, in zebra finches, PV-positive projection neurons are present in large numbers in the song control motor pathway of males but not females [43] and in the related Bengalese finch, PV gene expression is higher in males than females in HVC, RA and Area X at 45 days posthatching and in adulthood [44]. We therefore recently started investigating the possible sex difference in PNN, PV and their association in the zebra finch brain. As these investigations were in progress, an article appeared indicating that PNN expression is higher in the HVC and RA of males as compared to females but that this sex difference was not present in nuclei of the anterior forebrain pathway, LMAN and Area X (or the corresponding part of the basal ganglia in females) that is largely implicated in song learning and song stability [45]. We therefore slightly reorganized our investigations with two goals in mind: 1) determine the reproducibility of the sex differences previously reported in the caudal but not rostral song control system and 2) assess their anatomical specificity by studying whether such differences are present in the song system only or also extend to auditory and visual areas where a dense, functionally significant expression of PNN has also been observed in mammalian species. This question is particularly relevant since previous work in other species showed that PNN regulate plasticity in multiple functional networks including those involved in vision, somatosensation, fear conditioning and spinal motor control [27,28].

## Material and Methods

### Animals and tissue collection

Six adult male and six adult female zebra finches (*Taeniopygia guttata*) from our own breeding colony were used for this study. Birds were over 160 days of age when their brain was collected, an age when song learning is entirely completed and the related brain plasticity is presumably reduced. They had been raised in a common aviary (2 x 2 x 2 m) with their parents and a large group (n = more or less 40–50 depending on breeding stage) of conspecifics). They had thus been exposed to extensive social interactions and tutor song. Throughout their life, birds were held on a 13L:11D dark/light cycle and they had food and water constantly available *ad libitum*.

Birds were captured and immediately anesthetized within less than 3 min with pentobarbital (0.05 ml Nembutal at 0.6 mg/ml). The heart was exposed and a blood sample was collected with a syringe from the left ventricle. Birds were then perfused with 200 ml phosphate buffer saline (PBS) followed by paraformaldehyde (4% in PBS) fixation. Brains were dissected out of the skull and postfixed for 24 hours in the same fixative. They were transferred into a 30% sucrose solution until they sank, frozen on dry ice and kept at -80°C until processed.

### Ethics statement

All experimental procedures were in agreement with the Belgian laws on animal experimentation and had been approved by the local Animal Care Committee (Commission d’Ethique de l’Utilisation des Animaux d’Expérience de l’Université de Liège; Protocol number 1396).

### Immunohistochemistry

Brains were cut on a cryostat at -20°C in the sagittal plane at 30 μm thickness and collected in 4 series of 3 wells for each hemisphere (120 μm between each section in one series). One series of sections from each bird was stained by double label immunohistochemistry for perineuronal nets and parvalbumin using procedures similar to those described in Balmer et al. (2009). Sections were blocked during 30 minutes in 5% Normal Goat Serum diluted in Tris buffered saline (TBS) with 0.1% Triton-X-100 (TBST). They were then incubated overnight at 4°C in a mixture of two primary antibodies diluted in TBST: a mouse monoclonal anti-chondroitin sulfate antibody (CS-56; C8035, 1:500, Sigma Aldrich) specific for the glycosaminoglycan portion of the chondroitin sulfate proteoglycans that are main components of the perineuronal nets (PNN) extracellular matrix and a polyclonal rabbit anti-parvalbumin (PV) antibody (ab11427, 1:1000, Abcam). These two antibodies were used and validated in two previous studies on the same species [8,45]. Bound primary antibodies were then visualized using fluorescent secondary antibodies diluted in TBST: a goat anti-mouse antibody coupled to Alexa fluor 488 (green labeling, 1:100, Invitrogen) and a goat anti-rabbit antibody coupled to Alexa fluor 546 (red labeling, 1:200, Invitrogen). Finally sections were mounted on slides using TBS with gelatin and coverslipped with the Vectashield mounting medium containing DAPI to stain all cell nuclei.

All sections were stained in 3 batches, each containing 2 males and 2 females. Statistical analysis revealed however no effect of the staining batch and this factor was not considered in subsequent analyses.

### Data analyses

Regions of interest (ROI) were visually detected based on the presence of PNN and PV staining. Multiple brain regions were clearly showing, even at low magnification, a dense PNN immunofluorescence compared to the rest of the brain. These nuclei included the song system nuclei (HVC, used as a proper name, see [12]; the robust nucleus of the arcopallium, RA; Area X of the basal ganglia in males, an equivalent area located at the same anatomical position with respect with nearby landmarks such as the lateral magnocellular nucleus of the anterior nidopallium and the lamina pallio-subpallialis but not discernible by itself was quantified in females; the lateral magnocellular nucleus of the anterior nidopallium, LMAN), 3 auditory areas (Field L; the dorsal lateral mesencephalic nucleus, MLd; the medial nucleus of the dorsolateral thalamus, DLM), and 3 visual areas (the nucleus rotondus, ROT; the nucleus spiriformis lateralis, SpL; the nucleus subpretectalis, SP).

One picture of each ROI for each marker (PNN, PV, and DAPI) was taken with the 40x objective of a microscope connected to a digital camera in the section where the ROI had the largest surface. One picture was collected for each bird and each hemisphere. Once the microscope was focused using the PNN staining, the 3 pictures with the 3 different filter sets were taken with the same focus and frame. Pictures included almost the entire ROI except for Area X, ROT, SpL, Field L and MLd that were larger than one 40x microscope field, in which case the field of view was centered in the ROI.

Pictures were taken with a Leica DMRB Fl. 100 microscope for fluorescence and a Leica DFC-480 color camera. The same settings (gain and exposure) were used for the same staining in a specific region for all subjects. Settings were different between ROI because some had a brightest background demanding a decrease in gain or exposure time. Pictures were adjusted for brightness and contrast with the Image J software (NIH, Research Service Branch) using the same settings for a given ROI in all subjects. Selected photomicrographs presented in this paper were additionally obtained by confocal microscopy on a Nikon A1R microscope (488 and 561 nm lasers).

The number of DAPI stained nuclei in the entire images were automatically counted using the “particle count” function of the Image J software. Parvalbumin-immunoreactive (PV-ir) cells and PNN were visually counted in the corresponding images. PV-ir cells were defined as round to ovoid red fluorescent filled structures. PNN were defined as more or less circular fluorescent green shapes (brighter than the background) surrounding a PV-ir cell or a DAPI-positive nucleus, as defined in the merged images (PV+PNN, PNN+DAPI) in Image J. We counted in this way, the total numbers of DAPI-positive, PV-ir cells and PNN as well as the numbers of PV-ir cells surrounded by PNN. These numbers were then used to calculate the percentage of DAPI cells that were PV-ir (%PV-ir cells) or were surrounded by PNN (%PNN cells), the percentage of PV-ir cells surrounded by PNN (%PV with PNN) and the percentage of PNN surrounding PV-ir cells (%PNN with PV) were also calculated, which provided a form of normalization of data for regional differences in cell densities.

Additionally, we finally measured in all PNN images the average intensity of the green fluorescence (Alexa 488). These measures were obtained with the Image J software on native pictures that had not been adjusted for brightness or contrast. Each pixel was assigned a grey value between 0 and 255 and the average brightness of the entire ROI was then obtained (PNN density). Pictures were then made binary and thresholded to highlight only surfaces that contained detectable immunofluorescence and the program calculated the percentage of the area covered by immunopositive material (PNN fractional area or % PNN area). These measures were taken while the observer was blind to the sex of the birds.

### Statistical Analysis

All data for the song control nuclei (absolute numbers of DAPI-positive cells, PV-ir cells, PNN and PNN+PV as well as their proportions and the PNN immunofluorescence intensity and PNN fractional areas as described in the previous section) were initially analyzed by three-way mixed models analyses of variance (3-way ANOVA; 2x2x3) with the sex of the birds (male-female) and staining batch (3 levels) as fixed factors and the brain hemisphere (left-right) as repeated measures factor. Since no effect of the staining batch could be detected, this factor was then excluded and data concerning song control nuclei as well as auditory and visual areas were analyzed by 2-way ANOVA (2x2) with sex as fixed factor and brain hemisphere as repeated factor.

In a few cases, some sections could not be quantified due to various technical problems thus reducing the number of available data points. Final sample sizes are presented in Table 1. In visual areas, the immunoreactive signals could not be analyzed for a number of subjects in one or the other hemisphere due to technical problems with the PV immunostaining. The analysis of lateralization in these regions thus had a limited power and since no left-right asymmetry had been detected in the song control and visual nuclei (see results below), these data, averaged for left and right side when they were both available, were simply analyzed by one-way ANOVA with sex as a factor.

**Table 1:** Sex differences in the numbers of DAPI positive cells, in numbers of parvalbumin-immunoreactive (PV-ir) cells, of perineuronal nets (PNN) and in PV-ir cells surrounded by PN (PV+PNN), and in the intensity and percentage of surface covered by chondroitin sulfate-immunoreactive material (PNN intensity and PNN fraction area) in 4 song control nuclei and 3 auditory and 3 visual areas of the zebra finch brain.

			Absolute cell numbers					Intensity & Area		
			DAPI	PV	PNN	PV+PNN	Sample Size	PNN intensity	PNN fraction area (%)	Sample Size
**SONG**	HVC	Male	304.41 (17.17)	16.08 (2.11)	9.33 (1.91)	5 (1.39)	n = 6	28.97 (1.92)	60.37 (1.17)	n = 4
**CONTROL**		Female	315.67 (21.75)	21.5 (4.49)	4.42 (0.61)	0.58 (0.32)	n = 6	17.33 (3.43)	62.74 (2.56)	n = 4
**NUCLEI**		F	0.25	0.93	12.34	19.43		7.73	2.48	
		p	0.626	0.357	**0.006** **	**0.001** ***		**0.032** *	0.166	
	RA	Male	204.67 (18.78)	30.58 (5.04)	10.33 (2.95)	7.5 (2.50)	n = 6	43.45 (4.6)	59.02 (0.94)	n = 3
		Female	242.17 (25.00)	18.00 (2.37)	6.50 (1.44)	2.08 (1.25)	n = 6	32.30 (2.68)	56.89 (1.48)	n = 4
		F	2.74	9.72	1.86	12.11		2.81	1.21	
		p	0.129	**0.011** *	0.203	**0.006** **		0.155	0.322	
	Area X	Male	203.92 (25.45)	19.50 (2.44)	5.00 (2.67)	1.33 (0.53)	n = 6	17.99 (2.34)	59.06 (1.27)	n = 4
		Female	214.75 (22.22)	22.17 (2.52)	3.00 (0.76)	1.83 (0.67)	n = 6	19.14 (1.68)	58.45 (1.21)	n = 4
		F	0.20	1.29	7.71	0.40		0.12	0.43	
		p	0.665	0.282	**0.020** *	0.540		0.737	0.537	
	LMAN	Male	251.42 (30.83)	29.58 (5.21)	3.42 (1.05)	2.83 (0.79)	n = 6	41.08 (2.00)	57.35 (1.59)	n = 4
		Female	261.50 (22.98)	50.33 (9.02)	3.00 (0.89)	1.17 (0.42)	n = 6	30.73 (3.53)	55.20 (2.12)	n = 4
		F	0.09	4.06	0.04	4.60		5.80	1.19	
		p	0.774	0.072 (*)	0.840	0.058 (*)		0.053 (*)	0.318	
**AUDITORY**	Field L	Male	243.17 (28.56)	27.58 (2.46)	5.58 (0.79)	4.92 (0.83)	n = 6	34.78 (5.47)	59.23 (1.25)	n = 6
**AREAS**		Female	279.70 (18.77)	30.50 (2.07)	5.20 (0.64)	4.20 (0.60)	n = 5	30.86 (1.35)	57.18 (1.83)	n = 6
		F	1.04	0.78	0.13	0.45		0.48	0.85	
		p	0.335	0.400	0.723	0.519		0.053 (*)	0.318	
	DLM	Male	244.80 (11.01)	44.80 (3.42)	5.10 (1.29)	4.60 (1.22)	n = 5	26.48 (2.04)	55.56 (1.86)	n = 6
		Female	271.10 (19.88)	46.80 (7.09)	7.50 (1.95)	7.20 (2.02)	n = 5	28.42 (4.28)	56.80 (1.10)	n = 6
		F	1.04	0.06	1.05	1.22		0.17	0.33	
		p	0.337	0.806	0.334	0.302		0.691	0.579	
	MLD	Male	264.00 (19.42)	11.88 (2.33)	12.88 (2.25)	2.63 (0.52)	n = 4	42.00 (6.98)	55.35 (0.92)	n = 6
		Female	261.50 (22.91)	13.17 (1.90)	11.83 (1.17)	4.00 (0.56)	n = 6	41.17 (2.88)	56.62 (1.03)	n = 6
		F	0.01	0.18	0.20	2.86		0.00	0.79	
		p	0.941	0.679	0.663	0.129		0.990	0.396	
**VISUAL**	ROT	Male	142.00 (10.18)	29.58 (4.39)	12.75 (2.34)	9.00 (2.09)	n = 6	23.66 (3.13)	54.04 (2.72)	n = 6
**AREAS**		Female	142.67 (9.59)	28.92 (3.96)	10.25 (1.91)	7.67 (1.50)	n = 6	27.17 (2.24)	52.95 (3.49)	n = 6
		F	0.00	0.04	0.80	0.30		1.05	0.09	
		p	0.957	0.853	0.391	0.593		0.330	0.768	
	SPL	Male	174.00 (16.55)	36.90 (5.17)	11.50 (1.16)	8.70 (0.84)	n = 5	23.51 (3.19)	52.66 (2.76)	n = 6
		Female	177.67 (19.92)	37.25 (3.96)	10.83 (1.28)	7.58 (0.79)	n = 6	25.07 (2.92)	49.35 (2.06)	n = 6
		F	0.06	0.01	0.36	2.18		0.19	0.38	
		p	0.813	0.927	0.563	0.173		0.672	0.551	
	SP	Male	238.50 (21.44)	35.42 (3.57)	20.25 (3.18)	18.08 (3.22)	n = 6	22.86 (2.57)	52.69 (3.69)	n = 6
		Female	217.42 (23.30)	38.50 (2.76)	21.17 (2.03)	18.50 (1.76)	n = 6	24.21 (2.76)	53.17 (3.40)	n = 6
		F	0.60	0.53	0.12	0.02		0.18	0.02	
		p	0.457	0.484	0.735	0.886		0.683	0.891	

In each case the table lists the mean + SEM (in parentheses) of values in males and females, followed by the associated F and p obtained in the corresponding ANOVA (see methods for detail of statistical analyses). The sample size in each case is indicated in a last column separately for all cell counts first and then for PNN intensity and fractional areas.

*** = p<0.001

** = p<0.01, = p<0.05 and

(*) = 0.10<p<0.05.

Overall no effect of lateralization and no interaction between brain side and sex was detected with only a few exceptions (significant effect of side for PNN area in RA only [p = 0.017]; significant interaction for number of PV-ir cells surrounded by PNN in area X [p = 0.032] and number of PNN around PV-ir cells in SP [p = 0.015]) that were actually less numerous than what would be excepted by chance (Type I error). The rest of this presentation will therefore focus exclusively on sex differences observed in the ANOVAs.

## Results

### Neuroanatomical distribution of PV and PNN

**Fig 1:**
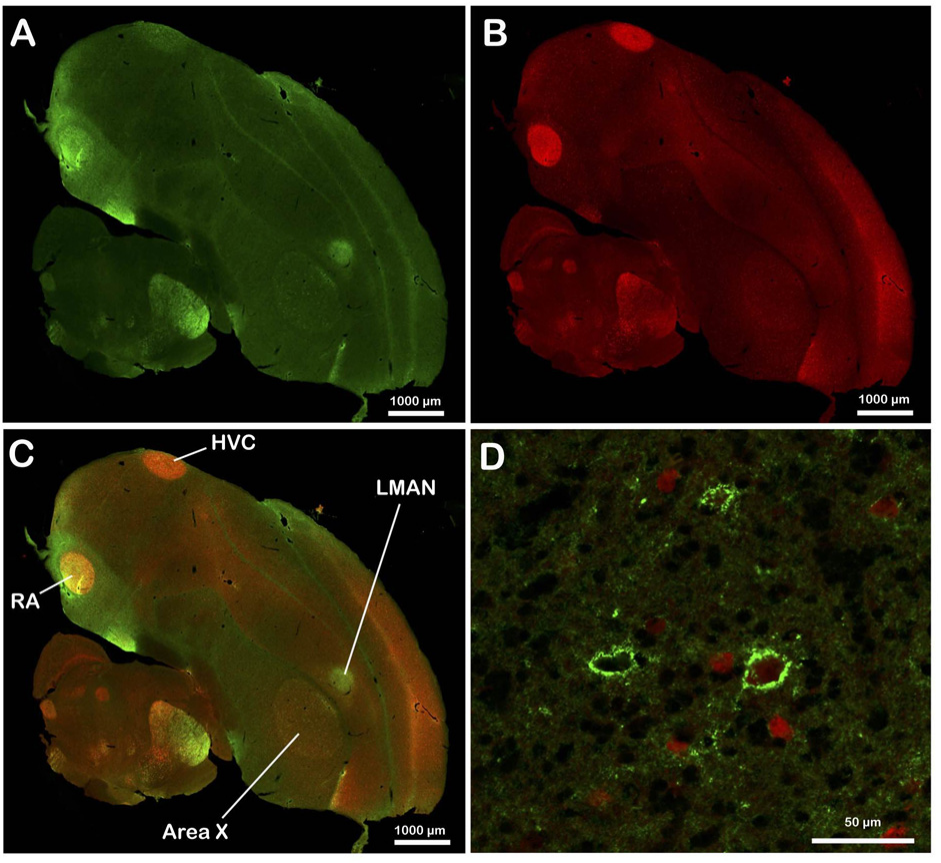
Representative photomicrographs illustrating the distribution of chrondroitin sulfate immunoreactivity and of parvalbumin-immunoreactive cells in parasagittal sections of the male zebra finch brain. Panels A through C illustrate at low magnification the chrondroitin sulfate (A) and parvalbumin (B) distribution as well as the overlay of the two signals (C). Panel D illustrates a few PNN surrounding or not parvalbumin-immunoreactive cells in Area X.

As previously reported [8], at low magnification the chondroitin sulfate-related green immunofluorescence (Alexa 488) was very heterogeneously distributed in the telencephalon, diencephalon, mesencephalon and brain stem (Fig 1A). A dense immunofluorescence namely labeled the song control nuclei (HVC, RA, area X and LMAN; Fig 1A and 1C) but also, in other parasagittal planes, auditory areas such as Field L, MLd, DLM, but not NCM or CMM, and visual areas including ROT, SpL and SP. All these nuclei were highlighted by a dense immunofluorescence as compared to the surrounding. At higher magnification, it could be observed that in many of these brain regions a large fraction of the immunofluorescent material was organized in brightly fluorescent rings specifically surrounding cells, the perineuronal nets (Fig 1D).

Similarly, inspection at low magnification of sections with filters visualizing the red Alexa 546 associated with parvalbumin identified dense clusters of immunopositive cells that specifically overlapped with the telencephalic song control nuclei (HVC, RA, Area X and LMAN) and with many other diencephalic and mesencephalic structures (Fig 1B and 1C). At higher magnification, this immunofluorescence was associated with labeled perikarya. Quite interestingly, a very substantial fraction (often more than half at first sight) of the PNN were surrounding cells that were themselves PV-immunopositive and this anatomical relationship was subsequently quantified (Fig 1D).

### Quantitative analyses

Quantitative analyses failed to detect significant differences in the total number of DAPI-positive cells between males and females in all brain areas that were analyzed (see Table 1). All sex differences detected for PNN and PV-ir cells and their relative numbers are thus not the result of general differences in cell densities and specifically concern these neurochemical markers.

### Song control nuclei

**Fig 2:**
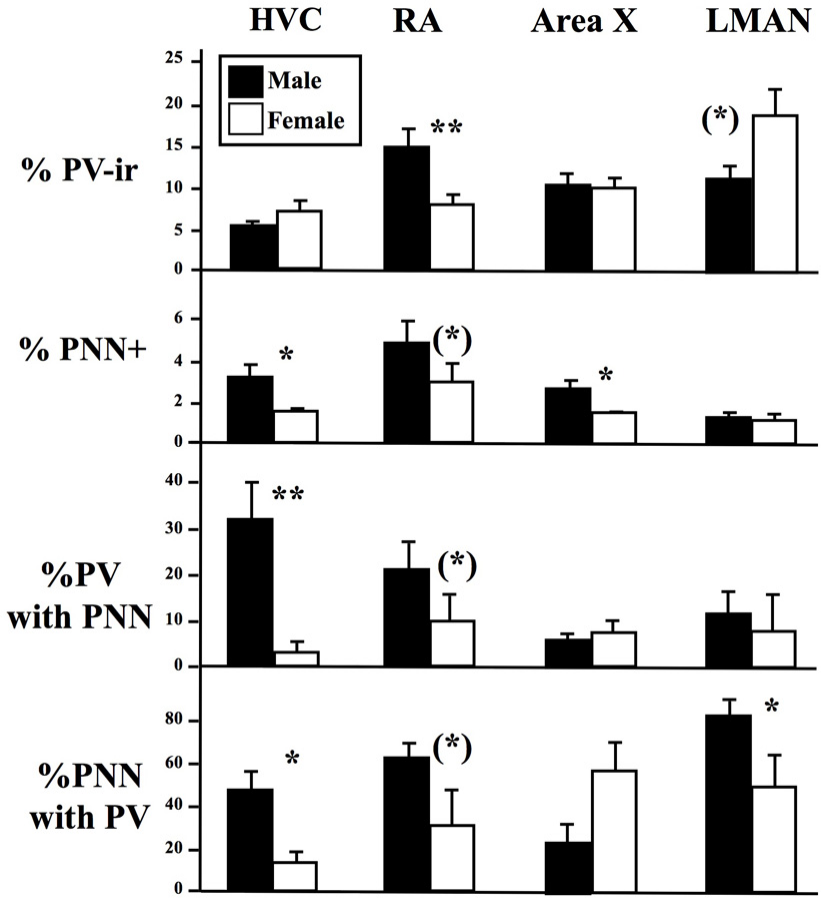
Sex differences in the percentage of DAPI cells that were immunoreactive for parvalbumin (%PV-ir cells) or were surrounded by perineuronal nets (%PNN cells), in the percentage of PV-ir cells surrounded by PNN (%PV with PNN) and in the percentage of PNN surrounding PV-ir cells (%PNN with PV) in 4 song control nuclei, HVC (used as a proper name), RA (robust nucleus of the arcopallium), Area X of the basal ganglia and LMAN (lateral magnocellular nucleus of the anterior nidopallium). The figure shows the mean ± SEM of data in males (black bars) and females (open bars). Numbers of data in each case are listed in Table 1. ** = p<0.01, * = p<0.05 and (*) = 0.10<p<0.05.

The four main telencephalic song control nuclei were clearly identified by dense populations of PV-ir cells associated with PNN (Table 1). Depending on the nucleus, 5 to 15% of the DAPI-positive cells expressed PV and 1 to 8% of them were surrounded by PNN. These two neurochemical markers were also frequently associated so that 3 to 30% of PV-ir cells were surrounded by PNN and conversely 12 to 87% of PNN surrounded PV-ir cells (these are extreme figures depending on the nucleus and sex; see Fig 2 and Table 1).

**Fig 3:**
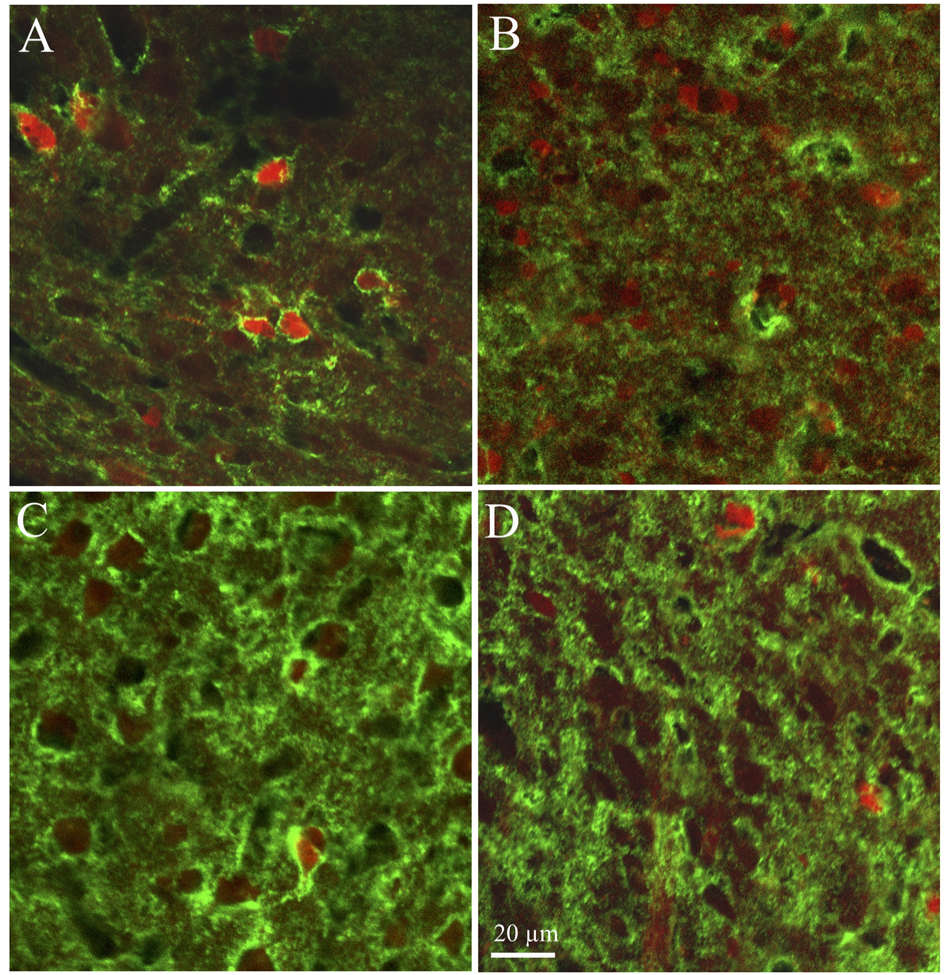
Representative photomicrographs illustrating the distribution of chrondroitin sulfate immunoreactivity (Green signal) and of parvalbumin-immunoreactive cells (red signal) as observed in in parasagittal sections of male (C, C) and female (B, D) zebra finch brains. Panels A_B illustrate differences in HVC, panels C-D concern RA. The magnification bar in D (20 μm) also applies to all other panels.

In HVC, PNN expression and its association with PV-ir cells was sexually differentiated. Males had a larger absolute number of PNN and of PV-ir cells surrounded by PNN (Table 1), a larger percentage of DAPI-positive cells surrounded by PNN (p = 0.011), of PV-ir surrounded by PNN (p = 0.005) and of PNN surrounding PV-ir cells (p = 0.004; see representative photomicrographs in Fig 3A and 3B).

Very similar trends were observed in RA with a more frequent association between PNN and PV-ir cells in males than in females (Fig 3C and 3D). However some of the differences that were significant in HVC were only associated here with statistical tendencies (p<0.10) and the only fully significant differences concerned the absolute number of PV-ir cells surrounded by PNN (Table 1) and the percentage of DAPI-positive cells expressing PV (p = 0.005).

The situation was different in Area X and LMAN, the two nuclei of the anterior forebrain pathway. Two significant differences only were detected in Area X: males had a larger total number of PNN (Table 1) and correlatively a larger percentage of DAPI cells surrounded by PNN (p = 0.023). Females had surprisingly a numerically larger percentage of PNN surrounding PV-ir cells but this difference was not significant (p = 0.10). In LMAN, one single sex difference was present: in males a larger percentage of PNN was surrounding PV-ir cells (p = 0.047). This difference was accompanied by one statistical trend pointing in the same direction: males tended to have a larger absolute number of PV-ir cells surrounded by PNN than females (Table 1). However females tended to have more PV-ir cells than males in absolute number (Table 1) and in percentage of DAPI-positive cells (p = 0.069).

Analysis of the overall PNN immunofluorescence intensity and fractional area covered by PNN immunofluorescence by the same statistical techniques (except that data from only 4 males and 4 females could be used in general with one male being excluded from the analysis of RA nuclei as a statistical outlier) identified few significant sex differences (Table 1): males had an overall denser PNN immunofluorescence intensity than females in HVC and a similar statistical tendency was observed in LMAN.

### Auditory areas

A high intensity of PNN fluorescence was located in three nuclei that are part of the auditory system, MLd, DLM and Field L.

**Fig 4:**
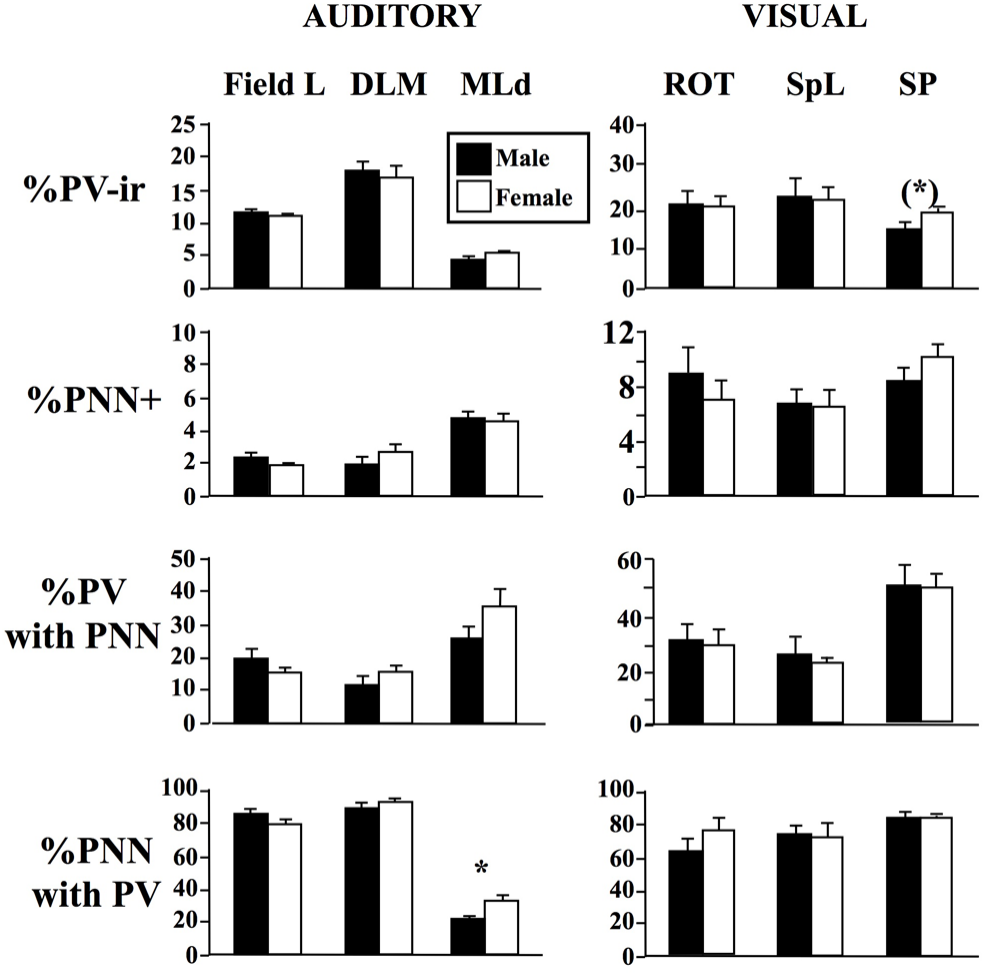
Sex differences in the percentage of DAPI cells that were immunoreactive for parvalbumin (%PV-ir cells) or were surrounded by perineuronal nets (%PNN cells), in the percentage of PV-ir cells surrounded by PNN (%PV with PNN) and in the percentage of PNN surrounding PV-ir cells (%PNN with PV) in 3 auditory areas (Field L; the dorsal lateral mesencephalic nucleus, MLd; the medial nucleus of the dorsolateral thalamus, DLM), and 3 visual areas (the nucleus rotondus, ROT; the nucleus spiriformis lateralis, SpL; the nucleus subpretectalis, SP). The figure shows the mean ± SEM of data in males (black bars) and females (open bars). Numbers of data in each case are listed in Table 1. * = p<0.05 and (*) = 0.10<p<0.05.

The same 10 dependent variables were analyzed in these three nuclei (See Table 1 and Fig 4). No sex difference was however observed in these nuclei with one exception: the percentage of PNN surrounding PV-ir cells was larger in females than in males in MLd (p = 0.049).

### Visual areas

Similarly, the same 10 dependent variables were collected from three visual areas (Table 1 and Fig 4) where a dense PNN immunoreactivity was observed, ROT, SpL and SP. No sex difference was however observed in these 3 brain regions with the potential exception that there was a statistical trend (p = 0.056) in SP for females to have a higher percentage of DAPI cells showing PV immunoreactivity compared to males.

## Discussion

This study demonstrates the existence in zebra finches of a major sex difference (males > females) affecting the number of PNN in HVC and to a smaller extent RA, the two main nuclei of the caudal forebrain pathway controlling song production. The difference concerned mostly and was by far more prominent for the PNN associated with cells expressing PV that are to a large extent local inhibitory neurons. In contrast, such differences were in general (see below for exceptions) not present in Area X and LMAN the two main nuclei of the anterior forebrain pathway. Because PNN have been implicated into the plasticity of visual areas in mammals [29,46,47], we extended these studies to investigate whether such sex differences would be present in the visual and also auditory pathways. A dense expression of material immunoreactive for chondroitin sulfate was detected in several nuclei of these sensory pathways and this material was often organized in perineuronal rings but quantification of these PNN did not reveal any sex difference with the exception that the percentage of PNN surrounding PV-ir cells in MLd was larger in females than in males, a sex difference in the opposite direction compared to what is seen in the caudal forebrain pathway of the song system. Interestingly there was almost no chondroidin sulfate-immunoreactive material in the secondary auditory area, NCM and CMM and no PNN could be detected in these structures. No sex difference was similarly observed in the visual areas that were investigated.

### Comparison with previous work

The distribution pattern of PNN and PV-ir cells in the song control nuclei observed here essentially matches and confirms results recently published on the same topic while our study was in progress [45]. This published study indeed also identified major sex differences in PNN numbers in HVC and RA but a lack of such differences in Area X and LMAN.

Three dependent variables were reported by Meyer and colleagues and can thus be directly compared with the present results: the percentage PV-ir cells, the percentage of cells surrounded by PNN, and the percentage PV-ir cells with PNN. With only one exception, similar numerical trends were always observed in both studies (males > females or females > males depending on the variable and nucleus) and limited differences between our results and those of Meyer et al. only concern the degree of statistical significance of a few results. Specifically, in HVC the % of PV-ir cells was larger in females than in males in both studies but the difference was significant in the study of Meyer and colleagues (p = 0,0014) but not here (p = 0,34). Conversely in RA this percentage was higher in males than in females in both studies but the difference was highly significant here (p<0.005) but less so in Meyer and colleagues (p = 0.043). The percentage of PV-ir cells surrounded by PNN in RA was larger in males than females in both studies but this difference was very significant in the study of Meyer and colleagues (p<0.001) and only marginal here (0.071)

The largest discrepancy between the two studies concerns the percentage of cells surrounded by PNN in Area X, which was sexually differentiated here (males>females, p = 0.023) but not at all in Meyer et al. (p = 0.76). Since Area X is the brain region where PNN are the clearest and can most easily be distinguished from the more diffuse immunoreactivity, it appears very unlikely that the discrepancy could relate to differences in counting efficiency. Area X is however a very large area and its equivalent structure in the female basal ganglia is not necessarily obvious. The difference between studies could thus in this case relate to the sampling of different brain subregions.

These limited differences could result from the use of different cell markers. Indeed, Meyer et al. used a Nissl stain to label all brain cells whereas we used a DAPI staining of cell nuclei. Absolute number of cells used as a basis for percentage calculation could thus differ. There might also exist subtle differences in staining intensity and counting methods that could produce these minor discrepancies but the high degree of reproducibility of the pattern found in song control nuclei is very reinforcing.

The present study also confirms earlier studies on PV-ir cells by showing that the absolute number and percentage of PV-ir cells in RA is larger in males than in females. One paper had indeed shown that in this nucleus PV-positive neurons projecting to motor neurons of the tracheosynringeal nucleus (nXIIts) are more numerous in males than in females [43]. The sex difference associated with a subset of neurons (those projecting to nXIIts) is thus carrying over to the entire population. In addition in the related Bengalese finch, the total number of cells expressing PV mRNA is higher in males than females in HVC, RA and Area X at 45 days post-hatching and in adulthood and the numbers of these cells per unit surface is higher in the HVC and RA of adult males [44]. Why these differences translate into sex differences in the numbers of cells expressing the protein in RA only and not in other nuclei (nucleus-specific regulation of translation, differential sensitivity of *in situ* hybridization and immunohistochemistry) remains unclear at present.

### Functional significance

The lower density of PNN in the female HVC and RA, the two song control nuclei of the motor pathway, has been suggested to reflect the fact that zebra finch females never sing [45]. Therefore these brain structures would maintain in females a greater degree of neuronal plasticity in contrast to males in which crystallization of song structure seems to be associated during ontogeny with an increase in PNN density that should limit potential changes in neuronal connectivity [8]. In contrast, since females learn like males to recognize conspecific songs (See introduction) and are probably able to update this information during their adult life, the anterior forebrain pathway that is implicated in song learning, should display no major sex difference in neural plasticity. The detailed quantification of PNN performed here in the song control nuclei is in general agreement with these ideas. Based on these speculations, one would then predict that in species where females sing either spontaneously (duetting birds such as tropical wrens; e.g. [48]) or after treatment with exogenous testosterone (e.g. canaries, *Serinus canaria* and European starlings, *Sturnus vulgaris*)[49–51], the sex difference identified in HVC and RA should no longer exist after the females have crystallized their song and we are currently testing this idea in canaries.

Somewhat to our surprise, we found here no major difference in PNN expression in the auditory and visual areas of the zebra finch brain. It might have been anticipated that males and females process auditory information in a different manner and this could be reflected in PNN local densities. The presence of estrogen receptors alpha and of aromatase has indeed been detected in the inner ear of zebra finches [52] and aspects of aromatase expression are sexually differentiated in auditory regions, in particular the NCM, of the zebra finch brain [53,54], raising the possibility of a control of auditory activity by circulating or locally produced sex steroids. This possibility is supported by a few studies of auditory brainstem responses (ABRs), the immediate output of the inner ear, recorded by electrophysiological techniques at the brainstem level. ABRs are far-field action potentials resulting from synchronous neural discharges along the ascending auditory pathway in response to the onset of auditory stimuli. They appear as a suite of peaks or waves representing the progressive propagation of neural auditory activity. These ABRs are changing seasonally in songbirds [55] and appear to be sexually differentiated in zebra finches (higher amplitude and shorter latency responses for waves 1 and 2 in females than in males)[56]. Another aspect of auditory responses to amplitude-modulated tones, the envelope following responses (EFRs), thought to reflect the strength of phase locking of the stimulus in the auditory nerve and possibly brainstem to the envelope, was also shown to differ between male and female cowbirds, *Molothrus ater* [57].

In addition, an abundant literature demonstrates that in mammals PNN limit plasticity in the visual system and develop following exposure to light. Following monocular light deprivation, plasticity is maintained in the “blind” hemisphere and PNN become established in much smaller amounts [46,47]. There is to our knowledge no direct evidence demonstrating sex differences in the activity of visual pathways in birds but such evidence exists in mammals [58]. Furthermore aromatase has been identified in fibers and perikarya of the visual system in quail [59]. Aromatase-immunoreactive fibers are also present throughout the visual system of neonate rats (optic tract, suprachiasmatic nucleus, lateral hypothalamus, geniculate nuclei, lateral posterior thalamic nucleus and all layers of the superior colliculus). Immunoreactive cell bodies were also seen in the superior colliculus, suprachiasmatic nucleus and intergeniculate leaflet [60,61]. Estrogen receptors have finally been identified in the developing rat cortex [62–64]. Furthermore sex differences were detected in the total dendritic length for three populations of cortical neurons within the visual cortex of the rat brain [65]. These observations therefore support the possibility of a differential control of visual activity by peripheral or centrally-synthesized estrogens.

Together all these data suggesting sex differences in the central processing of auditory and visual information lead us to investigate whether there are in parallel sex differences in plasticity maintained by PNN in the corresponding brain regions. Chondroitin sulfate immunoreactivity was indeed identified in these areas and was even condensed in PNN in at least three nuclei of each sensory pathway. Interestingly, no PNN were detected in the secondary auditory area NCM suggesting that this brain region playing a critical role in storing/decoding/interpreting conspecific song [42,66] retains a large degree of plasticity. However even in nuclei where PNN were abundant, no sex difference could be detected neither in their number nor in overall chondroitin sulfate immunoreactivity with the possible exception that the percentage of PNN associated with PV immunoreactive cells was higher in females than in males in the auditory nucleus MLd (p = 0.049). Given the number of comparisons that were performed and the proximity of the associated probability with the classically admitted critical level for accepting statistical significance, this difference would become non significant after Bonferroni correction.

This lack of sex difference might seem surprising at first sight but is not necessarily incompatible with previous work. Indeed although the development of PNN in the mammalian visual system has clearly been shown to be activity-dependent [28,31,67], one study indicated that in young chicken the organization of the extracellular matrix into PNN is not importantly dependent on light activation in the mesencephalic, diencephalic and telencephalic visual centers [68]. Whether this difference between species reflects a general difference between birds and mammals is not known at present but if it was the case, this might contribute to explain the lack of sex differences observed here. It must also be mentioned that although (neuro)anatomical and, to a limited extent, functional sex differences have been identified in the auditory and visual systems they do not necessarily imply differences in neuroanatomical plasticity. Furthermore even if a sex difference in plasticity is present, it could be reflected in other morphological markers independent of PNN, such as (polysialyated) Neural Cell Adhesion Molecule PSA-NCAM or dendritic spines.

## Supporting Information

S1 Dataset Individual values of all measures that were collected and analyzed in this article. This excel table contains all raw data that were use in the present paper organized in 10 individual sheets, one for each nucleus considered.
(XLS)
